# The global burden of traumatic amputation in 204 countries and territories

**DOI:** 10.3389/fpubh.2023.1258853

**Published:** 2023-10-20

**Authors:** Bei Yuan, Dong Hu, Suxi Gu, Songhua Xiao, Fei Song

**Affiliations:** Department of Orthopaedics, Beijing Tsinghua Changgung Hospital, School of Clinical Medicine, Tsinghua University, Beijing, China

**Keywords:** amputation, disability study, global burden of disease, incidence, years lived with disability

## Abstract

**Background:**

Traumatic amputation leads to disability and imposes a heavy health burden. This study aims to explore the current status and temporal trends of the global burden of traumatic amputation according to sex, age, amputation site, cause, and reginal level of social development.

**Methods:**

The data were extracted from the Global Burden of Diseases (GBD) Study 2019. Prevalence, incidence, years lived with disability (YLDs) and corresponding age-standardized rate were compared. Estimated annual percentage change (EAPC) was applied to reflect trends in age-standardized rates over a specific period. Spearman rank test and curve fitting methods were used to analyze the relationship between disease burden and Socio-Demographic Index (SDI).

**Results:**

Globally, the incidence and prevalence number of traumatic amputation increased from 11.37 million and 370.25 million in 1990, to 13.23 million and 552.45 million in 2019, with a raise of 16.4 and 49.2%, respectively. But the age-standardized incidence rate (ASIR) (EAPC = −0.56; 95%CI, −0.72 to −0.41) and age-standardize prevalence rate (ASPR) (EAPC = −0.63; 95%CI, −0.74 to −0.52) declined during this period. The YLDs count also increased by 39.2% globally (from 5.28 million to 7.35 million), while the age-standardize YLDs rate (ASYR) decreased by an average of 1.00% per year (95% CI, −1.10 to −0.90) from 1990 to 2019. The incidence, prevalence, and YLDs rate of traumatic amputation continue to increase with age. Traumatic amputations were most common in the fingers, while unilateral lower limb amputation caused the greatest burden of disability. ASIR and SDI were positively correlated (*ρ* = 0.442, *p* < 0.001), while ASYR and SDI were not significantly correlated (*ρ* = −0.030, *p* = 0.669), and EAPC in ASYR and SDI were negatively correlated (*ρ* = −0.275, *p* < 0.001). Exposure to mechanical forces and falls were the leading causes of traumatic amputation.

**Conclusion:**

Despite the declining trends in ASIR, ASPR, and ASYR, the incidence, prevalence, and YLDs counts of traumatic amputation have increased significantly worldwide, especially in the older adults population. With the population aging, targeted health policies are needed to address the increasing global burden of traumatic amputations in the future.

## 1. Introduction

Amputation causes disability, severely impairs human mobility and physical function, and has imposed a significant global health economic burden over the past several decades (1). The leading cause of amputation varies from region to region, peripheral vascular disease and diabetes have become the most important causes of amputation in developed countries (2, 3), which can be attributed to their escalating prevalence and the significant burden imposed on patients (4–6). However, in developing countries, trauma is still the main cause of amputation (7, 8).

Traumatic amputation is a disabling treatment option as a last resort when the limb suffers from mechanical injury, frostbite, burn, and electric injury, which leads to irreversible blood supply loss and cannot be repaired or treated with a huge cost but less function than artificial limb, and when serious complications threaten life (9, 10). Any accidental injury resulting in the loss of a limb or other bodily appendage is medically defined as traumatic amputation. Along with economic development and increased vehicle density, the number of traumatic amputations resulting from road traffic accidents in developing countries is increasing (11). In developed countries, the burden of traumatic amputation in the older adults is not negligible due to the aging of the population. In the United States, approximately 12% of emergency department visits for older adults patients suffered traumatic amputation (12). Traumatic amputation often requires long periods of rehabilitation, severely affecting the life quality of patients, while placing a heavy burden on health systems (13, 14). Despite this, the paucity of epidemiological studies on traumatic amputation suggests that it remains largely neglected.

Country-specific studies on epidemiological trends in traumatic amputations have been published in the past (11, 14, 15). However, very few studies have focused on the intercountry and global prevalence of traumatic amputations (1). The Global Burden of Diseases (GBD) Study is a global-level collaborative research project that produces consistent and transparent estimates of the burden of disability and mortality for most diseases and injuries by systematically integrating all available data (16). In this study, we used GBD Study 2019 to obtain the number of prevalence, incidence, years lived with disability (YLDs), age-standardized prevalence rate (ASPR), age-standardized incidence rate (ASIR), and age-standardized YLDs rate (ASYR) to investigate and assess epidemiological trends in traumatic amputation and to analyze etiologic components and associated factors influencing trends to provide evidence for health resource and policy adjustments.

## 2. Methods

### 2.1. Data source

To explore the current status and temporal trends of traumatic amputation, we extracted data from the GBD Study 2019.^1^ Estimates of disease and injury in the GBD study are based on multiple sources, including information from insurance claims, household surveys, censuses, civil registration, hospital records, disease registries, National Health Service records, satellite imaging, cause-of-death detection and reporting, and high-quality literature (16–18). The inclusion criteria for this study encompassed a diagnosed traumatic amputation, complete data on incidence, prevalence, and YLDs, as well as a time frame spanning from January 1990 to December 2019. The exclusion criteria involved cases where the data value for incidence, prevalence, or YLD was zero, unclear cause documentation, and an unknown age range. Finally, we obtained the data of incidence, prevalence, YLDs, and corresponding age-standardized rates (ASRs) based on sex, age, and cause for all patients with traumatic amputation in 204 countries and 21 regions. YLDs were defined as the sum of years of survival for any short-or long-term health loss due to amputation based on the disability-weighted severity.

### 2.2. Definitions

Traumatic amputation is defined using the WHO International Classification of Diseases codes 9th revision (ICD 9, 885.0–885.1, 0.0–886.1, 887.0–887.7, 895.0–895.1, 896.0–896.3, 897.0–897.7) and 10th revision (ICD 10, S48.0–S48.1, S48.9, S58.0–S58.1, S58.9, S68.0–S68.4, S68.8–S68.9, S78.0-S78.1, S78.9, S88.0–S88.1, S88.9, S98.0–S98.4, T05.0–T05.6, T11.6) in GBD Study 2019. The incidence of traumatic amputation is defined as the number of new cases of traumatic amputation in a given population during a given period. The prevalence of traumatic amputation is defined as the total number of new and old cases of traumatic amputation in a given population, over a given period. To compare the epidemiological differences of traumatic amputation in different regions and populations, ASRs were obtained by giving different weights to crude rates according to age composition. ASIR, ASPR, and ASYR were all directly available from the GBD database. The Socio-Demographic Index (SDI) is a summative assessment of the overall level of social development based on educational attainment, fertility rate, and *per capita* income (16). It is a geometric mean on a scale from 0 to 1, with higher values representing higher levels of social development.

### 2.3. Data processing and modeling

The GBD database researchers performed correction and standardized modeling on data registered from hospitalization records, emergency outpatient records, insurance claims, and injury surveillance based on ICD codes to estimate the prevalence, incidence, and YLD of traumatic amputations. Injuries in GBD study include two dimensions: the cause of injury, which refers to violence that acts directly on the body, and the nature of injury, which refers to the resulting consequences on the body. A cause-property matrix is created by dual coding of cause and nature of injury to assess the onset and prevalence of a given injury. DisMod-MR 2.1, a Bayesian meta-regression tool, was used to assess the incidence of each nature of injury and to enforce consistency in disease prevalence, incidence, and mortality rates. A detailed description of the estimation methods for injury in the GBD study 2019 has been published previously (16).

### 2.4. Statistical analysis

The number of prevalence, incidence, YLDs, ASPR, ASIR, and ASYR obtained from the GBD database is expressed as a value with a 95% uncertainty interval (UI). We applied the estimated annual percentage change (EAPC) to reflect trends in ASR over a specific period. We assumed that the natural logarithm of prevalence was linearly related to time variation and fitted by the regression model ln (ASR) = *α* + *β*x + *ε*, where x as the independent variable denotes calendar year. The value of EAPC and its 95% confidence interval (CI) can be calculated by the formula 100 × (exp(*β*) − 1). Spearman rank test and Curve fitting methods were used to analyze the relationship between disease burden and correlates such as SDI. All statistical analyzes and figure plotting were performed using R software (version 4.2.3). *p*-values less than 0.05 were regarded as statistically significant.

## 3. Results

### 3.1. Incidence and prevalence of traumatic amputation

Globally, there were 13.23 (95% UI, 11.05 to 15.91) million new cases of traumatic amputation with an ASIR of 171.23 (95% UI, 142.86 to 205.92) per 100,000 population in 2019 (Table 1). In comparison, the incidence number was 11.37 (95% UI, 9.4 to 13.9) million and the ASIR was 211.45 (95% UI, 175.21 to 257.06) per 100,000 population in 1990. From 1990 to 2019, the number of incidences increased by 16.4% but the ASIR decreased by 19.0% and showed a decreasing trend (EAPC = −0.56; 95%CI, −0.72 to −0.41). The prevalence of traumatic amputation showed the same pattern, with an increase in prevalence number from 370.25 million in 1990 to 552.45 million in 2019, but a decline in ASPR from 7,850.68 per 100,000 population in 1990 to 6,811.89 per 100,000 population in 2019 with an average annual decline of 0.63% (95% CI: −0.74 to −0.52%). At the gender level, ASIR and ASPR for traumatic amputations in men remained about twice as high as in women in both 1990 and 2019. There was a decreasing trend in ASIR and ASPR for both genders, and the decreasing trend was greater for males (Male: EAPC of ASIR = −0.66; EAPC of ASPR = −0.73; Female: EAPC of ASIR = −0.35; EAPC of ASPR = −0.46) (Table 1).

**Table 1:** The incidence and prevalence of traumatic amputation in 21 regions, with EAPC from 1990 and 2019.

location	Incidence					Prevalence				
	1990 Incidence No. × 10^3^ (95% UI)	1990 ASIR per 100,000 (95% UI)	2019 Incidence No. × 10^3^ (95% UI)	2019 ASIR per 100,000 (95% UI)	1990–2019 EAPC of ASIR (95% CI)	1990 Prevalence No. × 10^3^ (95% UI)	1990 ASPR per 100,000 (95% UI)	2019 Prevalence No. × 10^3^ (95% UI)	2019 ASPR per 100,000 (95% UI)	1990–2019 EAPC of ASPR (95% CI)
*Global*	11,367.40 (9,360.00 to 13,923.33)	211.45 (175.21 to 257.06)	13,232.85 (11,050.34 to 15,912.95)	171.23 (142.86 to 205.92)	−0.56 (−0.72 to −0.41)	370,253.34 (339,886.58 to 403,340.33)	7,850.68 (7,235.11 to 8,531.81)	552,448.08 (506,944.61 to 602,232.83)	6,811.89 (6,255.87 to 7,428.38)	−0.63 (−0.74 to −0.52)
*Sex*										
Male	7,706.77 (6,364.18 to 9,483.99)	281.96 (233.57 to 343.49)	8,593.84 (7,148.04 to 10,396.48)	221.41 (184.08 to 267.73)	−0.66 (−0.80 to −0.52)	247,740.07 (227,848.57 to 269,826.22)	10,731.87 (9,896.47 to 11,699.38)	359,511.91 (331,402.89 to 390,500.99)	9,025.65 (8,328.89 to 9,795.83)	−0.73 (−0.82 to −0.63)
Female	3,660.63 (2,988.60 to 4,571.73)	138.30 (113.84 to 170.89)	4,639.01 (3,852.11 to 5,684.33)	119.33 (98.71 to 146.49)	−0.35 (−0.54 to −0.16)	122,513.27 (111,170.96 to 135,301.64)	5,107.33 (4,662.30 to 5,610.38)	192,936.18 (175,476.83 to 213,945.83)	4,681.31 (4,245.93 to 5,215.38)	−0.46 (−0.59 to −0.32)
*Region*										
Andean Latin America	96.51 (76.03 to 125.58)	238.32 (188.68 to 306.52)	111.30 (90.18 to 140.34)	172.81 (140.11 to 217.07)	−0.78 (−1.06 to −0.50)	2,298.88 (2,070.38 to 2,600.50)	7,485.93 (6,845.51 to 8,273.56)	4,427.66 (4,042.07 to 4,856.93)	7,157.62 (6,543.45 to 7,838.67)	−0.25 (−0.29 to −0.22)
Australasia	133.47 (102.65 to 177.29)	672.76 (518.40 to 893.88)	170.75 (133.54 to 222.02)	640.09 (490.28 to 841.97)	−0.24 (−0.30 to −0.18)	5,715.89 (5,161.90 to 6,420.65)	26,185.88 (23,612.14 to 29,538.78)	8,731.50 (7,895.27 to 9,809.43)	25,156.61 (22,610.80 to 28,649.88)	−0.20 (−0.26 to −0.14)
Caribbean	68.46 (55.89 to 85.97)	187.56 (154.56 to 233.72)	98.53 (79.71 to 124.17)	210.03 (168.90 to 264.31)	0.86 (−0.57 to 2.30)	2,276.43 (2,091.18 to 2,481.85)	7,248.10 (6,669.45 to 7,865.26)	4,549.05 (4,126.98 to 5,046.87)	9,162.92 (8,300.26 to 10,181.56)	0.99 (0.87 to 1.12)
Central Asia	201.78 (161.72 to 255.16)	278.64 (224.14 to 351.10)	242.66 (193.45 to 308.42)	253.99 (202.41 to 321.98)	−0.86 (−1.31 to −0.41)	6,453.35 (5,898.35 to 7,076.74)	10,923.38 (10,025.87 to 11,914.75)	9,441.07 (8,607.67 to 10,369.27)	10,328.20 (9,441.59 to 11,321.10)	−0.22 (−0.28 to −0.16)
Central Europe	704.12 (556.63 to 904.21)	583.96 (462.44 to 753.32)	533.66 (417.63 to 684.90)	514.41 (400.44 to 671.64)	−0.65 (−0.80 to −0.49)	28,749.69 (26,141.50 to 31,526.62)	21,367.59 (19,398.60 to 23,465.14)	28,832.22 (26,200.06 to 31,763.90)	19,689.71 (17,759.60 to 21,842.45)	−0.25 (−0.33 to −0.18)
Central Latin America	441.94 (356.25 to 558.93)	253.68 (206.21 to 315.56)	502.21 (401.22 to 630.58)	201.51 (160.46 to 252.94)	−0.08 (−0.40 to 0.25)	13,419.80 (11,941.30 to 15,188.00)	10,063.93 (9,120.62 to 11,143.21)	21,596.95 (19,518.46 to 24,048.66)	8,535.18 (7,724.02 to 9,502.30)	0.26 (−0.01 to 0.52)
Central Sub-Saharan Africa	87.99 (66.03 to 120.74)	147.52 (112.78 to 197.95)	147.03 (119.84 to 181.30)	107.70 (89.71 to 129.82)	−2.44 (−4.27 to −0.58)	2,024.13 (1,673.41 to 2,557.15)	4,745.10 (4,090.11 to 5,721.52)	5,876.71 (4,455.57 to 8,095.17)	5,996.79 (4,626.13 to 8,220.25)	0.84 (0.56 to 1.13)
East Asia	996.56 (826.20 to 1,213.84)	82.80 (69.04 to 100.50)	1,597.82 (1,322.96 to 1,905.80)	101.01 (83.48 to 122.34)	0.34 (−0.01 to 0.68)	34,513.03 (31,651.54 to 37,648.88)	3,088.60 (2,844.45 to 3,367.71)	64,238.01 (59,462.78 to 69,865.74)	3,435.77 (3,177.79 to 3,728.98)	−0.06 (−0.40 to 0.27)
Eastern Europe	1,124.81 (905.38 to 1,410.43)	507.42 (407.36 to 639.36)	777.31 (626.94 to 972.15)	390.18 (314.41 to 489.38)	−0.97 (−1.11 to −0.83)	46,537.73 (42,411.65 to 50,948.06)	18,445.43 (16,790.64 to 20,178.24)	38,636.25 (35,399.04 to 42,356.06)	14,585.40 (13,287.80 to 16,046.90)	−0.91 (−1.00 to −0.81)
Eastern Sub-Saharan Africa	991.31 (563.02 to 1,658.36)	479.64 (277.22 to 793.21)	516.32 (426.18 to 633.28)	123.41 (103.35 to 149.43)	−2.95 (−4.15 to −1.73)	9,174.59 (7,267.36 to 12,599.39)	6,380.76 (5,301.04 to 8,146.19)	19,630.53 (15,805.82 to 25,894.14)	6,661.05 (5,351.78 to 8,763.65)	0.05 (−0.08 to 0.18)
High-income Asia Pacific	536.74 (427.59 to 681.28)	308.96 (245.90 to 391.61)	502.68 (404.62 to 628.42)	284.06 (223.14 to 363.55)	−0.43 (−0.55 to −0.31)	22,454.96 (20,402.55 to 24,691.16)	11,557.90 (10,483.19 to 12,736.66)	28,838.09 (26,258.46 to 31,643.66)	11,080.86 (10,023.40 to 12,340.81)	−0.25 (−0.39 to −0.12)
High-income North America	849.26 (679.65 to 1,073.72)	300.43 (238.60 to 384.45)	968.44 (798.51 to 1,165.62)	250.15 (203.61 to 310.19)	−1.16 (−1.49 to −0.83)	35,477.71 (32,328.69 to 38,972.26)	11,386.79 (10,367.73 to 12,554.96)	41,858.68 (38,562.10 to 45,370.41)	9,033.78 (8,280.15 to 9,887.31)	−1.55 (−2.02 to −1.07)
North Africa and Middle East	857.29 (705.00 to 1,050.70)	241.15 (200.40 to 293.00)	1,356.65 (1,060.27 to 1,765.55)	220.80 (172.88 to 288.42)	1.39 (0.75 to 2.03)	22,983.03 (18,920.48 to 28,997.91)	8,314.76 (7,042.55 to 10,207.25)	52,336.95 (42,913.54 to 66,190.11)	8,909.77 (7,354.36 to 11,254.90)	0.14 (0.02 to 0.26)
Oceania	6.39 (5.33 to 7.83)	98.32 (83.08 to 118.82)	13.37 (11.12 to 16.56)	101.45 (85.31 to 123.62)	−0.45 (−1.40 to 0.51)	170.37 (157.32 to 184.44)	3,556.88 (3,302.99 to 3,833.37)	438.48 (399.26 to 482.99)	4,087.39 (3,738.31 to 4,487.52)	0.44 (0.36 to 0.51)
South Asia	1,732.40 (1,444.98 to 2,097.20)	162.67 (137.41 to 194.57)	2,684.84 (2,241.10 to 3,250.75)	151.85 (127.84 to 182.48)	−0.38 (−0.69 to −0.07)	46,709.41 (43,118.85 to 50,612.29)	5,591.23 (5,180.04 to 6,026.38)	92,312.34 (85,200.78 to 99,687.15)	5,542.12 (5,127.88 to 5,974.68)	−0.13 (−0.20 to −0.06)
Southeast Asia	727.69 (595.42 to 894.93)	152.35 (126.17 to 185.72)	805.63 (663.51 to 985.10)	120.37 (99.63 to 146.60)	−0.65 (−1.22 to −0.07)	20,723.87 (18,512.28 to 24,366.05)	5,397.27 (4,894.30 to 6,167.65)	34,735.59 (31,429.72 to 39,018.21)	5,030.52 (4,568.35 to 5,619.94)	−0.34 (−0.44 to −0.25)
Southern Latin America	181.41 (142.72 to 235.25)	363.06 (285.38 to 469.69)	226.25 (178.12 to 292.66)	346.40 (272.39 to 447.67)	−0.38 (−0.48 to −0.28)	6,658.04 (6,063.68 to 7,304.08)	13,909.51 (12,685.48 to 15,239.97)	9,920.23 (9,052.14 to 10,920.99)	13,453.62 (12,261.50 to 14,858.62)	−0.31 (−0.40 to −0.22)
Southern Sub-Saharan Africa	69.24 (56.26 to 85.90)	128.78 (105.17 to 158.51)	86.15 (70.87 to 103.61)	108.48 (89.43 to 130.10)	−0.65 (−0.76 to −0.53)	2,120.68 (1,930.93 to 2,379.76)	5,084.23 (4,669.99 to 5,607.10)	3,124.94 (2,865.79 to 3,439.56)	4,289.93 (3,938.51 to 4,705.96)	−0.57 (−0.67 to −0.47)
Tropical Latin America	297.39 (241.08 to 369.24)	192.08 (156.04 to 235.63)	352.83 (288.69 to 432.12)	156.91 (128.37 to 193.41)	−0.69 (−0.78 to −0.60)	9,242.73 (8,465.38 to 10,168.01)	7,283.08 (6,709.34 to 7,972.17)	14,347.72 (13,152.14 to 15,739.51)	5,876.95 (5,379.03 to 6,432.46)	−0.81 (−0.92 to −0.71)
Western Europe	1,018.97 (821.31 to 1,278.48)	272.46 (218.64 to 347.68)	1,033.53 (829.54 to 1,304.35)	246.89 (194.06 to 318.12)	−0.53 (−0.61 to −0.46)	46,972.63 (42,647.92 to 52,155.26)	10,445.17 (9,456.80 to 11,611.59)	54,560.77 (49,311.17 to 60,726.51)	9,718.09 (8,699.21 to 10,924.72)	−0.45 (−0.54 to −0.36)
Western Sub-Saharan Africa	243.69 (192.69 to 315.02)	122.99 (98.72 to 157.21)	504.86 (408.81 to 632.46)	110.56 (90.86 to 136.00)	−0.27 (−0.67 to 0.12)	5,576.40 (5,111.71 to 6,082.51)	3,997.10 (3,704.82 to 4,297.30)	14,014.36 (12,547.67 to 15,845.57)	4,182.18 (3,792.81 to 4,685.25)	0.15 (0.07 to 0.23)

No., number; UI, uncertainty interval; ASIR, age-standardized incidence rate; ASPR, age-standardized prevalence rate; EAPC, estimated annual percentage change; CI, confidential interval.

At the regional level, South Asia had the highest incidence number (2.68 million, 95% UI, 2.24 to 3.25 million) and prevalence number (92.31 million, 95% UI, 85.20 to 99.69 million) of traumatic amputations, while Australasia had the highest ASIR (640.09, 95% UI, 490.28 to 841.97 per 100,000 population) and highest ASPR (25,156.61, 95% UI, 22,610.80 to 28,649.88 per 100,000 population) in 2019. East Asia had the lowest ASIR (101.01, 95% UI, 83.48 to 122.34 per 100,000 population) and lowest ASPR (3,435.77, 95% UI, 3,177.79 to 3,728.98 per 100,000 population) for traumatic amputations in 2019. The only region where ASIR increased was North Africa and Middle East, with an average annual increase of 1.39% (95% UI, 0.75 to 2.03%). ASIR in the rest of the region was trending downward or stable, with the Eastern Sub-Saharan Africa region showing the largest trend of ASIR decline, with an average annual decline of 2.95% (95% UI, −4.15% to −1.73%). ASPR declined in most regions, with High-income North America showing the largest decline of 1.55% (95% UI, −2.02% to −1.07%) per year. Caribbean had the largest upward trend in ASPR, with an average annual increase of 0.99% (95% UI, 0.87 to 1.12%) (Table 1).

**Figure 1:**
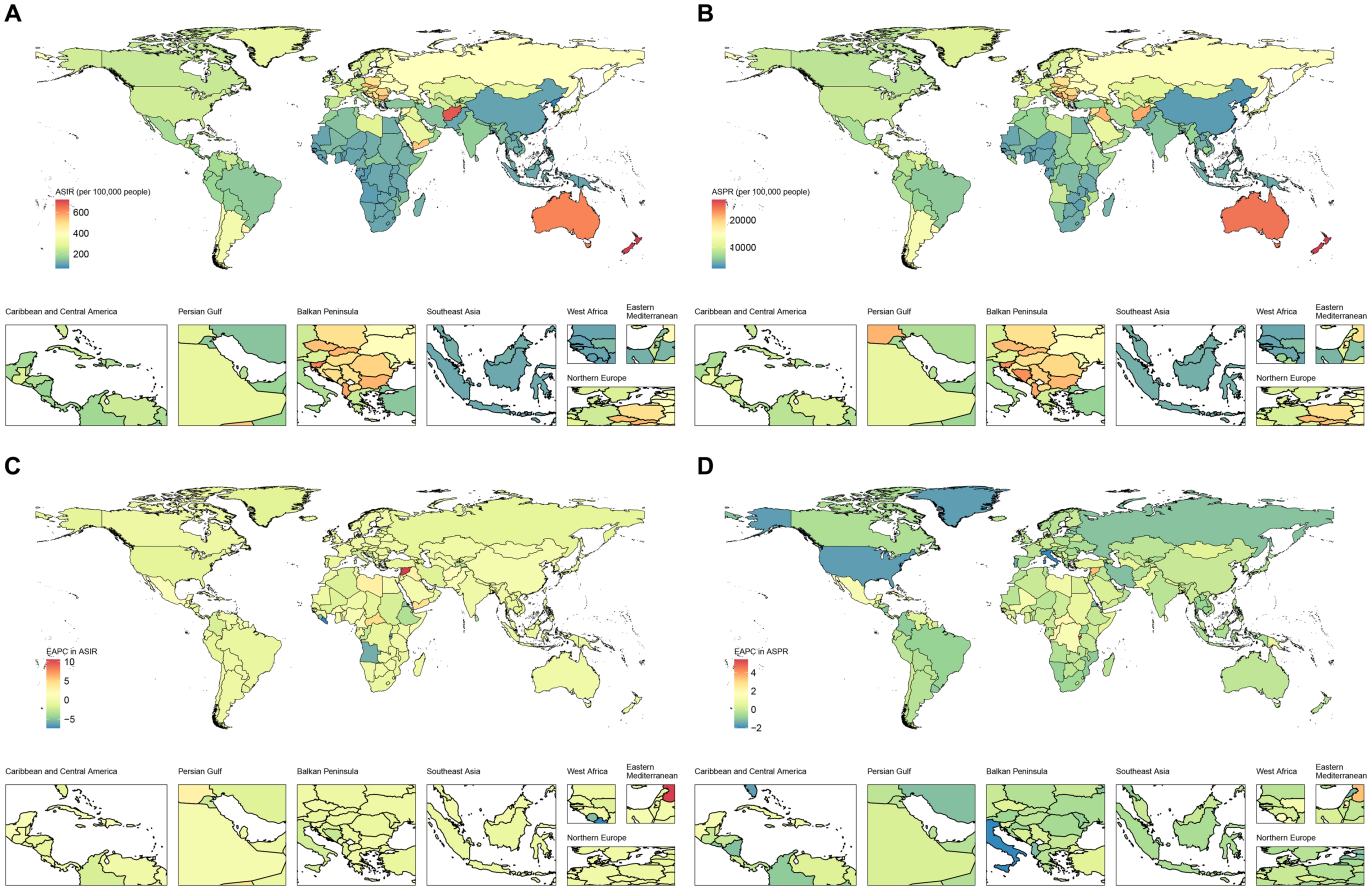
Geographical distribution of traumatic amputation in 204 countries and territories. **(A)** Geographical distribution of age-standardized incidence rate (ASIR) for traumatic amputations in 2019. **(B)** Geographical distribution of age-standardized prevalence rate (ASPR) for traumatic amputations in 2019. **(C)** Estimated annual percentage change (EAPC) in ASIR for traumatic amputations from 1990 to 2019. **(D)** EAPC in ASPR for traumatic amputations from 1990 to 2019.

At the country level, India had the highest number of incidence (2.22 million, 95% UI, 1.85 to 2.70 million) and prevalence (75.60 million, 95% UI, 69.66 to 81.97 million) in 2019, followed by China. Figures 1A,B show the ASIR and ASPR of traumatic amputations in 204 countries worldwide in 2019, respectively. New Zealand had the highest ASIR (722.04, 95% UI, 566.88 to 937.81 per 100,000 population) and ASPR (27,535.86, 95% UI, 24,803.25 to 31,180.66 per 100,000 population), while North Korea had the lowest ASIR (62.96, 95% UI, 53.00 to 76.02 per 100,000 population) and ASPR (2,316.99, 95% UI, 2,167.23 to 2,486.66 per 100,000 population) in 2019. Figures 1C,D show the trends in ASIR and ASPR for traumatic amputations in 204 countries worldwide from 1990 to 2019, respectively. Syrian Arab Republic has the largest upward trend in ASIR with an average annual increase of 10.59% (95% CI, 6.91 to 14.40%). Liberia had the largest decline in ASIR, with an average annual decline of 7.31% (95% CI, −10.08% to −4.45%). Burundi has the largest upward trend in ASPR with an average annual increase of 5.44% (95% CI, 4.05 to 6.84%). Italy had the largest decline in ASPR, with an average annual decline of 2.04% (95% CI, −2.52% to −1.55%).

### 3.2. Disability burden of traumatic amputation

Table 2 shows the temporal trends of the burden of disability due to traumatic amputation in 21 regions of the world. The global burden of disability in 2019 was 7.35 (95% UI, 4.77 to 11.29) million YLDs, with an ASYR of 90.68 (95%UI, 58.86 to 139.16) per 100,000. The global ASYR for traumatic amputations decreased by an average of 1.00% per year (95% CI, −1.10 to −0.90) from 1990 to 2019. Both YLDs and ASYR in males were approximately 1.6 times higher than those in females. At the regional level, South Asia had the highest number of YLDs (1.73 million, 95% UI, 1.23 to 2.41 million), while Australasia had the highest ASYR (207.59, 95% UI, 107.61 to 378.81 per 100,000) in 2019. The upward trend in ASYR was most pronounced in the Caribbean, with an average annual increase of 1.16% (95% CI, 0.83 to 1.50%), while the downward trend was most pronounced in East Asia, with an average annual decrease of 1.93% (95% CI, −2.42 to −1.44).

**Table 2:** The YLDs and ASYR of traumatic amputation in 21 regions, with EAPC from 1990 and 2019.

location	1990 YLDs, No. × 10^3^ (95% UI)	1990 ASYR per 100,000 (95% UI)	2019 YLDs, No. × 10^3^ (95% UI)	2019 ASYR per 100,000 (95% UI)	1990–2019 EAPC of ASYR (95% CI)
*Global*	5,282.09 (3,558.70 to 7,888.92)	113.92 (76.91 to 169.18)	7,354.91 (4,766.52 to 11,285.18)	90.68 (58.86 to 139.16)	−1.00 (−1.10 to −0.90)
*Sex*					
Male	3,278.22 (2,174.99 to 4,972.35)	143.95 (95.96 to 217.16)	4,469.32 (2,840.34 to 6,953.01)	112.49 (71.59 to 174.55)	−1.04 (−1.12 to −0.95)
Female	2,003.86 (1,383.83 to 2,911.03)	84.96 (58.79 to 122.50)	2,885.58 (1,910.00 to 4,307.57)	69.45 (45.84 to 103.81)	−0.94 (−1.07 to −0.81)
*Region*					
Andean Latin America	30.64 (20.74 to 46.21)	103.37 (70.46 to 152.88)	47.75 (29.70 to 77.76)	77.79 (48.38 to 126.09)	−1.11 (−1.16 to −1.07)
Australasia	47.12 (24.75 to 85.98)	215.67 (112.86 to 394.81)	73.26 (38.75 to 132.40)	207.59 (107.61 to 378.81)	−0.20 (−0.26 to −0.14)
Caribbean	29.06 (19.18 to 44.37)	94.44 (62.78 to 143.34)	59.49 (38.31 to 90.70)	119.99 (77.04 to 183.17)	1.16 (0.83 to 1.50)
Central Asia	79.61 (51.80 to 122.24)	137.43 (90.42 to 209.50)	103.51 (64.82 to 167.03)	114.58 (72.16 to 183.73)	−0.71 (−0.75 to −0.67)
Central Europe	314.11 (197.53 to 504.88)	232.07 (145.67 to 375.13)	259.66 (141.42 to 455.47)	173.33 (92.44 to 309.30)	−1.04 (−1.15 to −0.92)
Central Latin America	191.75 (129.47 to 285.88)	150.53 (103.21 to 219.96)	255.84 (162.21 to 405.10)	101.66 (64.62 to 160.52)	−0.43 (−0.71 to −0.15)
Central Sub-Saharan Africa	33.89 (22.91 to 50.95)	89.51 (63.48 to 126.80)	85.87 (54.64 to 136.33)	95.49 (62.90 to 146.24)	0.20 (−0.03 to 0.42)
East Asia	766.74 (553.20 to 1,059.74)	71.60 (52.31 to 97.95)	976.37 (617.95 to 1,473.96)	51.83 (32.82 to 78.59)	−1.93 (−2.42 to −1.44)
Eastern Europe	563.17 (360.48 to 879.08)	220.46 (140.82 to 345.13)	397.69 (232.37 to 662.00)	146.3 (84.38 to 247.76)	−1.69 (−1.87 to −1.51)
Eastern Sub-Saharan Africa	156.71 (106.68 to 236.43)	123.10 (87.41 to 176.24)	315.74 (210.45 to 480.75)	114.68 (77.95 to 171.99)	−0.41 (−0.53 to −0.29)
High-income Asia Pacific	234.36 (137.89 to 392.78)	120.42 (70.81 to 202.77)	297.60 (170.08 to 501.29)	108.79 (60.69 to 188.04)	−0.42 (−0.56 to −0.28)
High-income North America	403.70 (235.97 to 656.35)	128.30 (74.47 to 210.92)	517.99 (312.04 to 818.54)	107.15 (63.44 to 172.92)	−1.24 (−1.67 to −0.81)
North Africa and Middle East	307.86 (187.44 to 519.26)	115.13 (73.55 to 185.51)	562.42 (324.83 to 991.20)	97.15 (56.68 to 170.07)	−0.77 (−0.88 to −0.67)
Oceania	2.93 (2.08 to 4.07)	66.25 (47.57 to 90.72)	7.39 (5.23 to 10.38)	73.65 (52.57 to 102.58)	0.35 (0.26 to 0.44)
South Asia	941.99 (679.71 to 1,279.16)	122.65 (89.03 to 165.31)	1,734.39 (1,231.84 to 2,416.74)	108.92 (77.63 to 150.46)	−0.53 (−0.67 to −0.39)
Southeast Asia	339.74 (237.97 to 495.05)	94.06 (66.87 to 133.02)	502.04 (338.96 to 756.60)	73.83 (50.21 to 109.94)	−0.96 (−1.03 to −0.89)
Southern Latin America	71.64 (45.00 to 115.02)	150.40 (94.73 to 241.07)	92.53 (53.55 to 157.67)	124.71 (71.70 to 213.64)	−0.81 (−0.88 to −0.74)
Southern Sub-Saharan Africa	36.96 (26.04 to 52.70)	93.25 (66.62 to 130.10)	52.24 (36.48 to 75.46)	73.75 (51.90 to 105.22)	−0.73 (−0.91 to −0.55)
Tropical Latin America	174.07 (123.49 to 244.32)	141.31 (100.94 to 196.31)	237.28 (160.79 to 346.39)	97.35 (66.05 to 141.99)	−1.31 (−1.41 to −1.22)
Western Europe	449.67 (250.77 to 775.60)	98.00 (54.07 to 170.95)	529.88 (294.33 to 910.70)	89.91 (49.24 to 156.12)	−0.48 (−0.57 to −0.39)
Western Sub-Saharan Africa	106.39 (77.06 to 147.00)	83.12 (60.62 to 112.27)	245.97 (173.94 to 347.27)	80.75 (57.99 to 110.93)	−0.12 (−0.20 to −0.04)

YLDs, years lived with disability; No., number; UI, uncertainty interval; ASYR, age-standardized YLDs rate; EAPC, estimated annual percentage change; CI, confidential interval.

**Figure 2:**
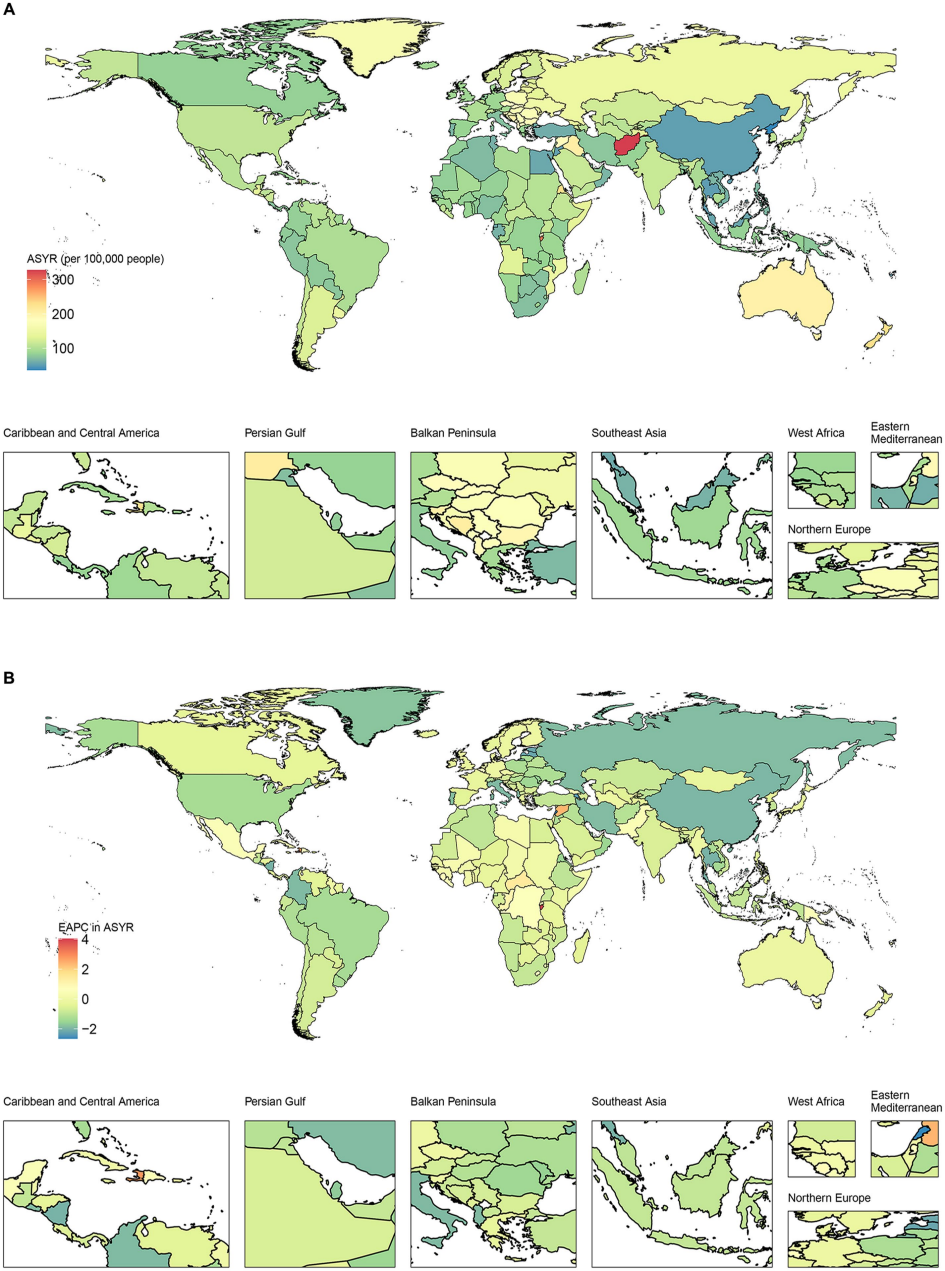
Geographical distribution of disability burden due to traumatic amputation in 204 countries and territories. **(A)** Geographical distribution of age-standardized years lived with disability rate (ASYR) for traumatic amputations in 2019. **(B)** Estimated annual percentage change (EAPC) in ASYR for traumatic amputations from 1990 to 2019.

Figure 2 shows the current status and trends of ASYR for traumatic amputation in 204 countries and territories worldwide. India had the highest number of YLDs at 1.45 (95% UI, 1.03 to 2.02) million in 2019, followed by China and the United States of America. Afghanistan had the highest ASYR at 327.13 (95% UI, 797.06 to 139.25) per 100,000 in 2019, followed by Burundi and Eritrea. Of the 204 countries and territories, 152 showed a decreasing trend in ASYR with Lebanon showing the largest decline of 2.66% (95% CI, −2.855 to −2.67%) per year, 29 showed an upward trend in ASYR with Burundi showing the most significant upward trend with an average annual increase of 4.04% (95% CI, 2.90 to 5.19%), while the ASYR remained stable in the rest.

### 3.3. Burden of traumatic amputation by age, gender and sites

**Figure 3:**
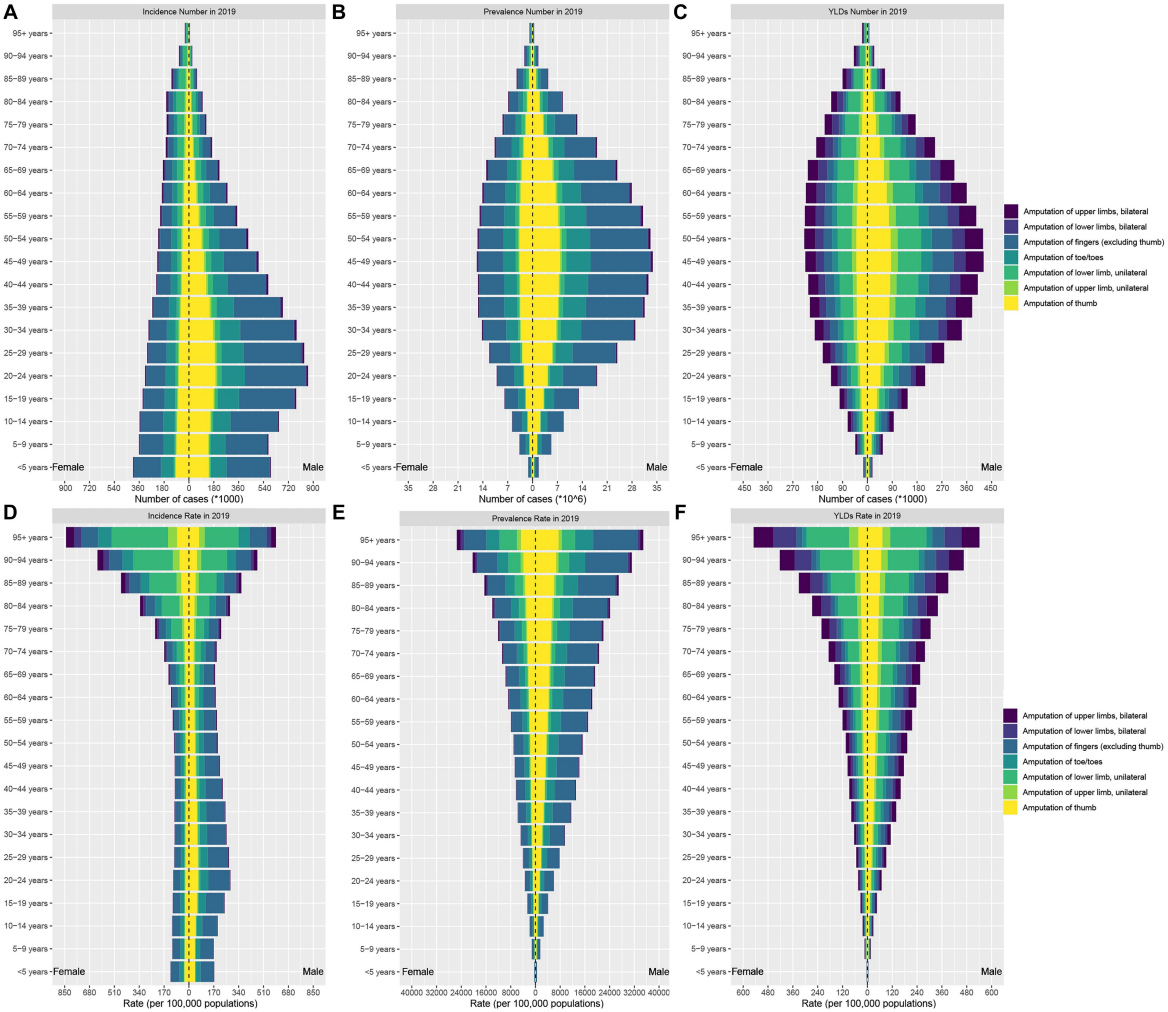
The incidence number **(A)**, prevalence number **(B)**, YLDs number **(C)**, incidence rate **(D)**, prevalence rate **(E)**, and YLDs rate **(F)** of traumatic amputation by age, sex, and sites in 2019. YLDs, years lived with disability.

The burden of traumatic amputation varies widely by age among males, with incidence count peaking at 20–24 years, and prevalence and YLDs counts peaking at 45–49 years (Figures 3A–C). The burden of traumatic amputation varies less by age among women, with the greatest incidence count occurring in patients younger than 5 years, the prevalence counts peaking at ages 45–49, and the YLDs counts peaking at ages 50–54. In contrast, the incidence, prevalence, and YLDs rates for both men and women increased with age and peaked in the 95 + age group (Figures 3D–F). At the gender level, as shown in Tables 1, 2, males had more incidence, prevalence, and YLDs counts than females. However, more female amputees were observed in the older adults population. The incidence counts of traumatic amputation are higher for women over 70 years old, YLDs counts are higher for women over 80 years old, and prevalence counts are higher for women over 85 years old (Figures 3A–C).

Amputation sites were classified as thumb, fingers (excluding thumb), toe/toes, unilateral upper limb, bilateral upper limb, unilateral lower limb, and bilateral lower limb for statistical analysis. As shown in Table 3, the incidence and prevalence of finger amputation (excluding thumb) were the highest, but unilateral lower limb amputation caused the greatest burden of disability (YLDs count, 1.60 million, 95%UI, 1.08 to 2.92 million) in 2019. ASIR, ASPR, and ASYR for all amputations declined between 1990 and 2019, except for bilateral lower extremity amputations, where ASIR remained stable. The prevalence count of finger amputation was highest in all age groups. In terms of incidence count, finger amputation ranks first among people under 75 years old, and unilateral lower limb amputation ranks first among people over 75 years old. In terms of disability burden, the number of YLDs with thumb amputation was highest among people under 40 years old, and the number of YLDs with unilateral lower limb amputation was highest among people over 40 years old.

**Table 3:** Global burden of traumatic amputation at different sites.

Sites	2019 Incidence No. × 10^3^ (95% UI)	2019 ASIR per 100,000 (95% UI)	1990–2019 EAPC of ASIR (95% CI)	2019 Prevalence No. × 10^3^ (95% UI)	2019 ASPR per 100,000 (95% UI)	1990–2019 EAPC of ASPR (95% CI)	2019 YLDs, No. × 10^3^ (95% UI)	2019 ASYR per 100,000 (95% UI)	1990–2019 EAPC of ASYR (95% CI)
Amputation of thumb	2,842.89 (1,962.99 to 4,085.32)	36.91 (25.43 to 53.06)	−0.63 (−0.77 to −0.48)	126,869.34 (116,070.16 to 139,596.37)	1,563.37 (1,429.29 to 1,720.00)	−0.76 (−0.89 to −0.62)	1,389.93 (681.59 to 2,615.94)	17.13 (8.39 to 32.19)	−0.75 (−0.89 to −0.62)
Amputation of fingers (excluding thumb)	5,896.90 (4,401.01 to 7,904.70)	76.64 (57.28 to 103.11)	−0.68 (−0.84 to −0.53)	261,071.06 (236,791.10 to 290,801.05)	3,218.84 (2,917.39 to 3,584.15)	−0.72 (−0.82 to −0.61)	1,191.98 (451.27 to 2,567.80)	14.70 (5.57 to 31.68)	−0.71 (−0.82 to −0.60)
Amputation of toe/toes	2,525.42 (1,776.35 to 3,456.40)	32.76 (22.96 to 44.97)	−0.52 (−0.80 to −0.24)	114,020.18 (99,372.53 to 138,697.23)	1,405.86 (1,223.77 to 1,708.75)	−0.43 (−0.52 to −0.34)	642.53 (257.07 to 1,415.89)	7.92 (3.17 to 17.48)	−0.42 (−0.52 to −0.33)
Amputation of upper limb, unilateral	321.26 (225.78 to 449.45)	4.07 (2.87 to 5.70)	−0.18 (−0.34 to −0.02)	9,607.20 (8,987.18 to 10,225.46)	118.40 (110.78 to 125.99)	−0.37 (−0.48 to −0.26)	464.25 (308.14 to 667.73)	5.72 (3.80 to 8.22)	−1.13 (−1.24 to −1.01)
Amputation of upper limbs, bilateral	302.56 (207.94 to 425.18)	3.84 (2.65 to 5.39)	−0.22 (−0.38 to −0.05)	9,282.56 (8,711.91 to 9,916.92)	114.41 (107.30 to 122.06)	−0.40 (−0.52 to −0.29)	1,216.58 (880.66 to 1,604.39)	15.00 (10.87 to 19.80)	−1.57 (−1.70 to −1.44)
Amputation of lower limb, unilateral	1,126.51 (877.82 to 1,417.06)	14.27 (11.12 to 17.95)	−0.15 (−0.25 to −0.05)	25,637.64 (23,398.23 to 29,393.72)	317.44 (289.75 to 363.99)	−0.26 (−0.30 to −0.21)	1,599.04 (1,079.52 to 2,292.38)	19.71 (13.26 to 28.25)	−1.01 (−1.10 to −0.93)
Amputation of lower limbs, bilateral	217.31 (135.44 to 346.02)	2.75 (1.71 to 4.35)	0.08 (−0.06 to 0.22)	5,960.95 (5,498.08 to 6,439.45)	73.57 (67.81 to 79.36)	−0.29 (−0.45 to −0.14)	850.60 (595.89 to 1,177.71)	10.49 (7.35 to 14.53)	−1.20 (−1.37 to −1.03)

No., number; UI, uncertainty interval; YLDs, years lived with disability; ASIR, age-standardized incidence rate; ASPR, age-standardized prevalence rate; ASYR, age-standardized YLDs rate; EAPC, estimated annual percentage change; CI, confidential interval.

### 3.4. Factors associated with the burden of traumatic amputation

**Figure 4:**
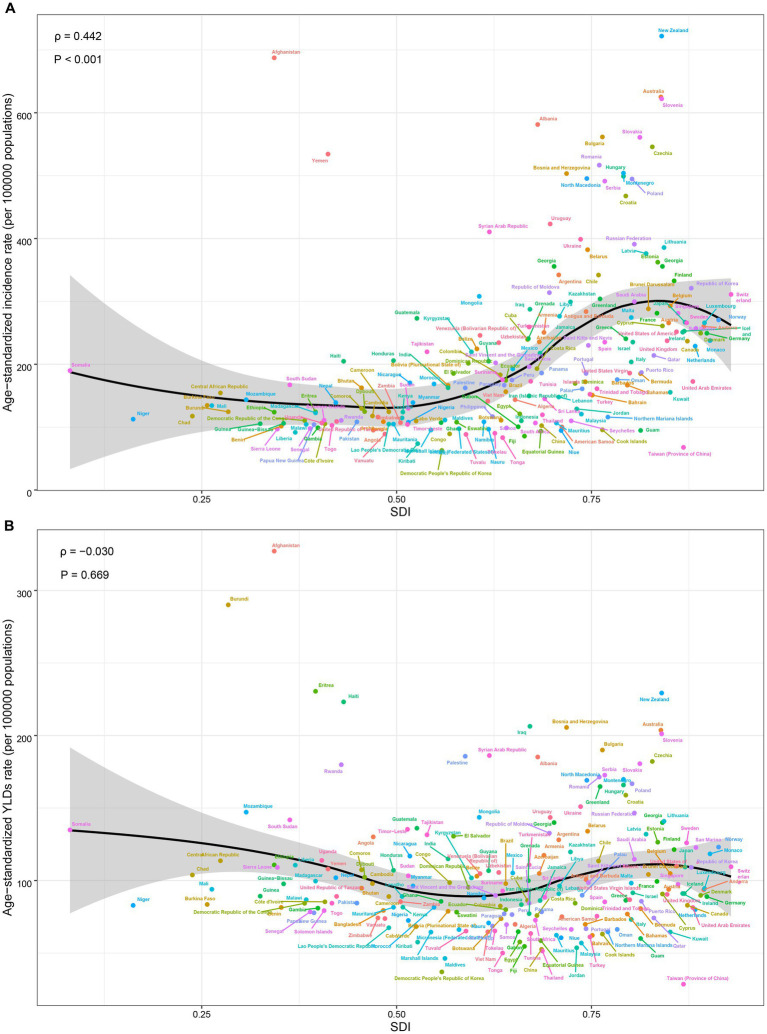
The correlation between the age-standardized incidence rate **(A)**, age-standardized years lived with disability rate **(B)** and socio-demographic index.

The correlation between ASR and SDI at the level of 204 countries and territories was shown in Figure 4. In general, ASIR and SDI were positively correlated (*ρ* = 0.442, *p* < 0.001), especially when SDI was between 0.5 and 0.8. Many developed countries such as New Zealand, Australia, and Slovenia have high ASIR (Figure 4A). In contrast, ASYR and SDI did not show a significant correlation (*ρ* = −0.030, *p* = 0.669) (Figure 4B).

**Figure 5:**
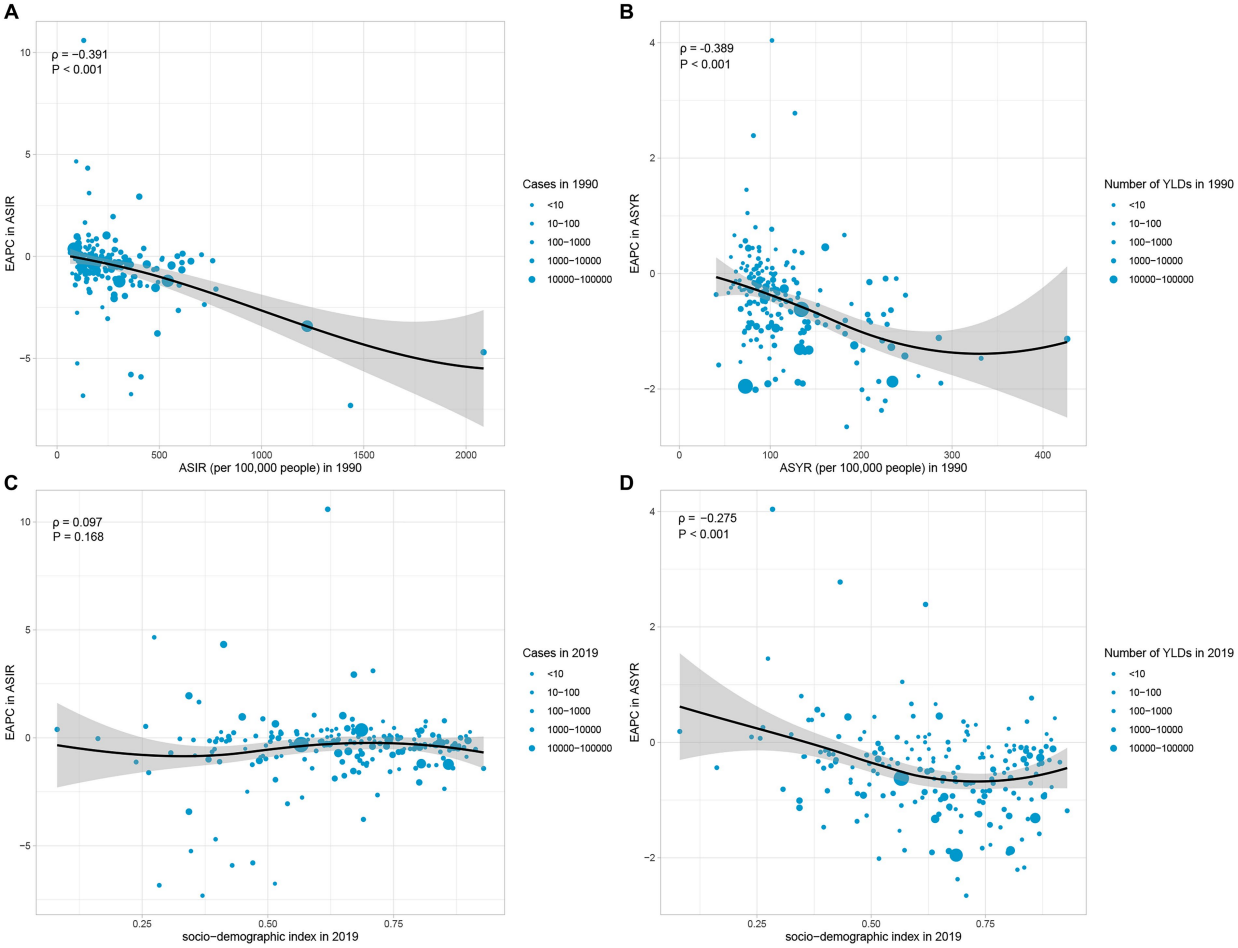
Factors associated with the EAPC. **(A)** The correlation between EAPC in ASIR and ASIR in 1990. **(B)** The correlation between EAPC in ASYR and ASYR in 1990. **(C)** The correlation between EAPC in ASIR and SDI. **(D)** The correlation between EAPC in ASYR and SDI. EAPC, estimated annual percentage change; ASIR, age-standardized incidence rate; ASYR, age-standardized years lived with disability rate; SDI, socio-demographic index.

We further explored the factors that influence the trend of ASR, including disease reservoir at baseline (reflected by ASR in 1990) and social development level (reflected by SDI). The results showed that there was a significant negative correlation between EAPC in ASIR and the baseline value of ASIR in 1990 (*ρ* = −0.391, p < 0.001) (Figure 5A). The same negative correlation was also observed in EAPC and ASYR (*ρ* = −0.389, p < 0.001) (Figure 5B). In the aspect of social development level, the EAPC in ASYR was negatively correlated with SDI (*ρ* = −0.275, *p* < 0.001), but there was no significant correlation between EAPC in ASIR and SDI (*ρ* = 0.097, *p* = 0.168) (Figures 5C,D).

### 3.5. Causes of traumatic amputation

**Figure 6:**
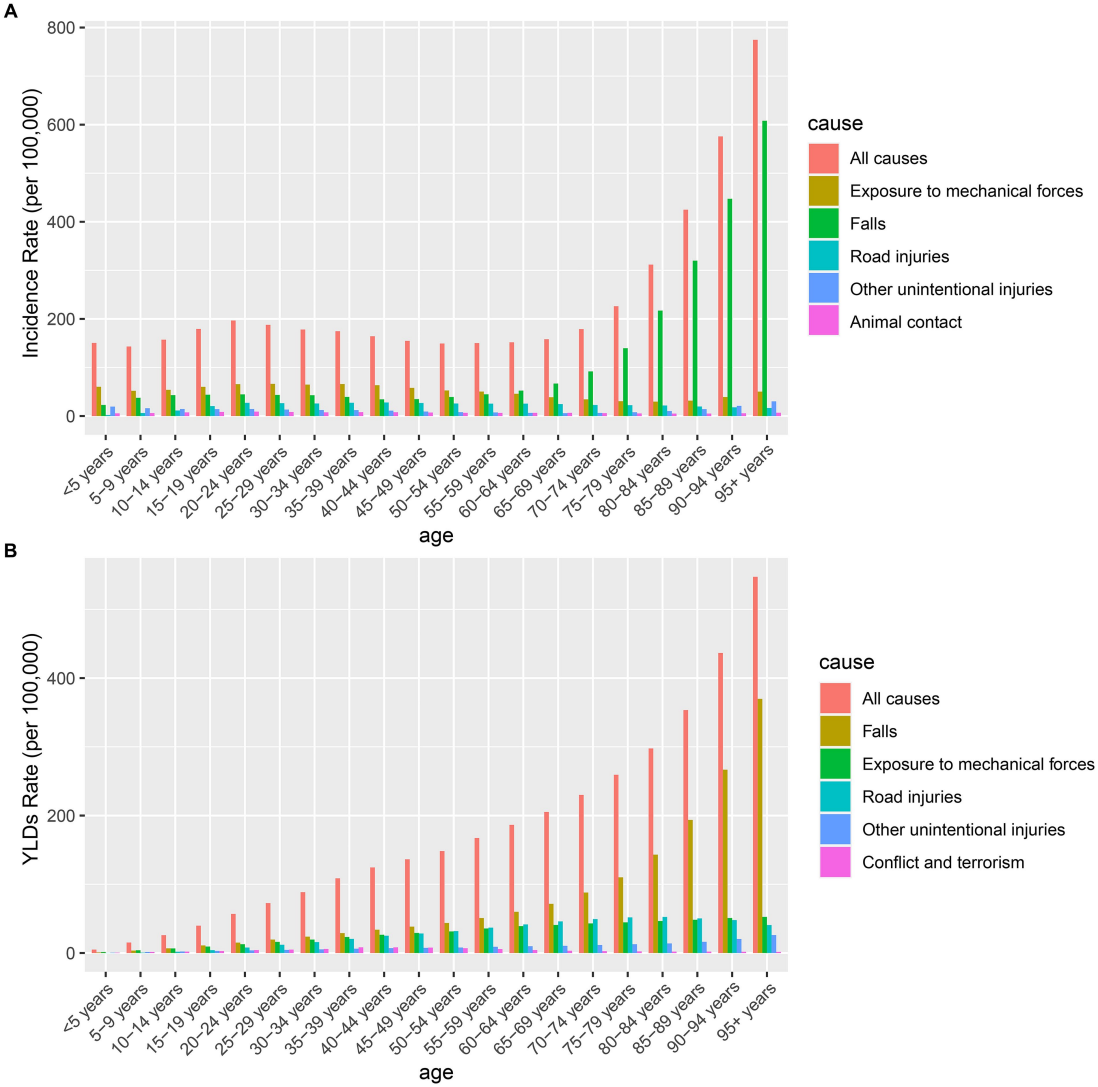
**(A)** The top five causes and all causes for the incidence rate of traumatic amputation in different age groups. **(B)** The top five causes and all causes for YLDs rate of traumatic amputation in different age groups. YLDs, years lived with disability.

Traumatic amputation had many causes in the GBD 2019, with the top five causes of incidence rate being exposure to mechanical forces, falls, road injuries, animal contact, and other unintentional injuries. Exposure to mechanical forces was the leading cause of the incidence of traumatic amputation, but the proportion of falls increases with age and becomes the leading cause in the over-60 age group (Figure 6A). The top five causes of YLDs rate were falls, exposure to mechanical forces, road injuries, other unintentional injuries, and conflict and terrorism. Except for people under 10 years of age, falls remain the leading cause of YLDs rate for traumatic amputation in all age groups (Figure 6B).

## 4. Discussion

Traumatic amputation is a health problem that places a heavy burden on global health systems yet is easily overlooked. Several studies have investigated epidemiological trends in traumatic amputations, but they tend to be limited to a single country or region, focus on amputations at a single site, or focus only on morbidity but ignore the burden of disability (1, 2, 14, 19). Based on literature and extensive data from 204 countries and territories, the present study provided a comprehensive estimate of global temporal and spatial trends in traumatic amputations by sex, age, amputation location, and cause, and analyzed factors associated with trends. The results showed that the incidence count, prevalence count and YLDs count of traumatic amputation increased significantly globally from 1990 to 2019, but after age standardization, ASIR, ASPR, and ASYR all showed a downward trend. Furthermore, the spatial distribution of the burden of traumatic amputation was not uniform, and there were great differences in the burden of amputation between different genders and different age groups. Baseline disease reservoir and social development were significantly associated with trends in amputation burden. Furthermore, we found exposure to mechanical forces and falls were the leading causes of traumatic amputation burden. This study effectively accounted for variability in age structure, eliminated the confounding effect of age across different geographic regions, enabled valid comparison of trends in traumatic amputation across these regions, and analyzed the causes of traumatic amputation among different age groups, providing compelling evidence to support the development of targeted prevention policies.

From 1990 to 2019, the incidence count, prevalence count and YLDs count of traumatic amputation increased by 16.4, 49.2, and 39.2% globally, respectively, but ASIR, ASPR, and ASYR decreased by 0.56, 0.63, and 1.00% per year on average, respectively. On the one hand, the global population increased by 45% from 5.3 billion to 7.7 billion between 1990 and 2019 (20, 21), which shows that the increase in absolute numbers is closely related to the sharp increase in population. On the other hand, the increasing life expectancy of the population and the aging of the population structure partly explain the increase in the number of prevalence and YLDs (20, 22). Despite the decline in ASYR, the increase in YLDs count can also place a burden on countries’ economies and health systems. Moreover, there were still as many as 29 countries and territories with ASYR rising year by year, which cannot be ignored.

In terms of gender and age, traumatic amputation mainly occurred in young men aged 20–60 years, with significantly lower ASIR, ASPR, and ASYR in women. This is partly attributed to the fact that males have more physical labor and a greater probability of violent injury in the social division of labor (23). In addition, more than 60% of traumatic amputations resulting from road traffic accidents are among young men (24). Notably, the number of cases and the burden of disability in older adults patients are greater among women. This is because falls are the leading cause of traumatic amputation in the older adults, and older women have a higher proportion of osteoporosis, leading to a greater chance of traumatic amputation (25). The incidence, prevalence, and YLDs rate of traumatic amputation continue to increase with age. In the context of the continuing aging of the population, the care of older persons requires greater attention.

Traumatic amputations were most common in the fingers, which is consistent with previous studies (12, 26). Fingers are most vulnerable to violent injury from mechanical tools such as saws and lawnmowers, and the lack of large muscle protection on the surface leads to increased chances of amputation (27, 28). Overall, unilateral lower limb amputation caused the greatest burden of disability. Specifically, the number of YLDs with thumb amputation was the highest under the age of 40, and the number of YLDs with unilateral lower limb amputation was the highest over the age of 40. The proportion of cases of unilateral lower limb amputation increased significantly with age, which indicated that preventive measures should be emphasized differently for different age groups. Amputation of the thumb also should be given sufficient attention, as it not only leads to functional impairment of the hand but also causes pathological psychological disorders that seriously reduce the quality of life of the patient (29).

Exposure to mechanical forces and falls were the two leading causes of traumatic amputations, followed by road injuries. Exposure to mechanical forces is responsible for the largest number of new cases of traumatic amputation, suggesting that future policies should focus on improvements in mechanical devices and occupational protection for workers. Traumatic amputations caused by falls contribute to the greatest burden of disability, especially in the older adults population. The incidence of falls in older adults and the health economic burden they create has increased rapidly over the past 20 years as life expectancy has increased (30, 31). Older adults people are prone to osteoporosis, and it is easy to cause lower limb fractures after falling, leading to surgery or even amputation (32, 33). More attention should be paid to the prevention of falls and the treatment of osteoporosis in the older adults in the future. In addition, road injuries were still an important cause of traumatic amputation, and it is reported that impact speed is closely related to the occurrence of traumatic amputation (34), so the speed limit and road traffic management still need to be improved in the future.

We found a positive association between the level of socioeconomic development and the incidence of traumatic amputation. High ASIR of traumatic amputation is observed in many high SDI countries such as New Zealand, Australia, Slovenia, and Slovakia, but only Yemen and Afghanistan have high ASIR in low SDI countries, which is caused by local wars. On the one hand, countries with high SDI have a higher level of industrialization, which leads to more mechanical force injuries and road traffic accidents. On the other hand, populations in high-SDI countries live longer and have a greater burden of osteoporosis and its associated fractures, which indirectly leads to more traumatic amputations (35). Nevertheless, the same level of ASYR as in the low SDI countries, and a greater downward trend in ASYR, were observed in the high SDI countries. Areas with higher levels of economic development have higher *per capita* health expenditures and provide more opportunities to improve health status (36), which does not reduce the incidence of traumatic amputation, but effectively reduces its disability burden.

This is the first study to systematically assess the global burden of traumatic amputation and its temporal trends. We report the incidence, prevalence, and YLDs of traumatic amputation based on sex, age, site, and cause to provide a comprehensive picture of the current situation. However, limitations still existed. First, the data for this study were extracted from the GBD, which was obtained by extrapolation from statistical models and inherently carries some uncertainty, especially in less developed regions. In addition, the description of the amputation site is not specific enough, such as lower limb amputation through the femur or tibia. Finally, the smallest region covered in this study was country, and epidemiological data from provinces and below were not involved. Even so, the GBD study provides a very rigorous computational approach and comprehensive data to estimate the global burden of traumatic amputations, which can guide the formulation of health policies and the allocation of medical resources.

## 5. Conclusion

From 1990 to 2019, global incidence count, prevalence count and YLDs count of traumatic amputation increased markedly, while the ASIR, ASPR, and ASYR all showed a downward trend. The spatial distribution of traumatic amputation burden was highly heterogeneous, with high ASIR observed in high-SDI countries, but equivalent ASYR levels as seen in low-SDI countries, as well as a greater ASYR decline trend noted in high-SDI countries. Men have a greater burden of amputation than women. Meanwhile, the incidence, prevalence, and YLDs rate of traumatic amputation continue to increase with age. Traumatic amputations were most common in the fingers, while unilateral lower limb amputation caused the greatest burden of disability. Exposure to mechanical forces was the leading cause of traumatic amputation, while falls were the leading cause of disability. The findings highlighted the need for targeted health policies to address the growing global burden of traumatic amputations.

## Data availability statement

Publicly available datasets were analyzed in this study. This data can be found at: https://vizhub.healthdata.org/gbd-results.

## Ethics statement

Ethical approval was not required for the study involving humans in accordance with the local legislation and institutional requirements. Written informed consent to participate in this study was not required from the participants or the participants’ legal guardians/next of kin in accordance with the national legislation and the institutional requirements.

## Author contributions

BY: Data curation, Investigation, Resources, Software, Visualization, Writing – original draft, Writing – review & editing. DH: Data curation, Formal analysis, Investigation, Software, Visualization, Writing – review & editing. SG: Conceptualization, Project administration, Supervision, Writing – review & editing. SX: Conceptualization, Methodology, Project administration, Supervision, Writing – review & editing. FS: Conceptualization, Investigation, Methodology, Project administration, Supervision, Writing – review & editing.

## Footnotes

^1^ https://vizhub.healthdata.org/gbd-results/
